# Syrian civil war and assessment of tuberculosis among Syrian refugees and local citizens in Mardin

**DOI:** 10.3389/fpubh.2025.1729829

**Published:** 2025-11-27

**Authors:** Barış Çil, Mehmet Kabak, Mehmet Sinan Bodur, Erkan Sanmak, Güldan Güneş, Yusuf Alakaş, Nicolai Savaskan, Hamdiye Turan, Hamza Oktay

**Affiliations:** 1 Department of Chest Diseases, Mardin Training and Research Hospital, Mardin, Türkiye; 2 Department of Pulmonary Diseases, Mardin Artuklu University Faculty of Medicine, Mardin, Türkiye; 3 Department of Pulmonology, Diyarbakır Gazi Yaşargil Training and Research Hospital, Diyarbakır, Türkiye; 4 Department of Microbiology, Mardin Training and Research Hospital, Mardin, Türkiye; 5 Mardin Provincial Health Directorate, Tuberculosis Dispensary, Mardin, Türkiye; 6 Department of Child Health and Diseases, Mardin Training and Research Hospital, Mardin, Türkiye; 7 Public Health Authority Gesundheitsamt Neukölln, Berlin, Germany; 8 Department of Pulmonary Diseases, Harran University Faculty of Medicine, Şanlıurfa, Türkiye

**Keywords:** tuberculosis, Syrian refugees, BCG, treatment outcomes, incidence, firth logistic, Mardin, Turkey

## Abstract

**Background:**

We compared tuberculosis (TB) characteristics and outcomes between Syrian refugees and local citizens in Mardin, Turkey (2016–2023), a border province with substantial population mobility.

**Methods:**

Retrospective, registry-based cross-sectional analysis of 491 patients (locals *n* = 456; refugees *n* = 35). Descriptive comparisons used *χ*^2^/Fisher (categorical) and Mann–Whitney U (age). Annual incidence per 100,000 used mid-year denominators (locals: ABPRS/NVI; refugees: DGMM/PMM and UNHCR). For outcomes with significant crude differences (treatment success, BCG scar, transferred-out), age- and sex-adjusted bias-reduced (Firth) logistic regression was applied; *p*-values from penalized likelihood-ratio (PLR) tests.

**Results:**

BCG-scar positivity was lower in refugees than locals (62.9% vs. 93.2%, *p* < 0.001). Microbiological confirmation remained below WHO targets in both groups. Crude treatment success was lower in refugees (68.6%) than locals (90.4%, *p* = 0.03), while transferred-out was higher (25.7% vs. 5.3%, *p* = 0.001). In adjusted Firth models including all cases, refugee status was associated with lower odds of success (aOR 0.224, 95% CI 0.103–0.488; PLR *p* < 0.001); after excluding transferred-out cases the association attenuated and was not significant (aOR 0.562, 95% CI 0.121–2.605; PLR *p* = 0.42). In pulmonary-only analyses, the association persisted (aOR 0.216, 95% CI 0.083–0.567; PLR *p* = 0.002). Refugee incidence dipped in 2020–2021 and rebounded in 2022–2023.

**Conclusion:**

Differences likely reflect operational barriers—especially transfers disrupting continuity—rather than intrinsic factors. Refugee-inclusive TB services with robust inter-provincial transfer tracking, patient navigation, and expanded bacteriological testing (notably for extrapulmonary disease) should be prioritized. Given the small refugee subgroup and denominator uncertainties, findings are hypothesis-generating.

## 1 Background

Wars deeply affect the social order, leading to long-term deterioration in social, cultural, and economic fields. Civil wars, in particular, increase social division, giving rise to acute and chronic crises at both individual and societal levels. The Syrian civil war, which started in 2011, caused the displacement of millions of people; this mass migration spread first within the country and then to neighboring countries. Turkey has been one of the countries most affected by this migration movement. As of June 2023, there are 3,351,582 Syrians registered under temporary protection in Turkey. 98% of this population lives in urban centers, while only 1.83% lives in temporary accommodation centers. With an average age of 22.5 years, this young population has special vulnerabilities in terms of access to health services and infectious disease control. This large-scale population movement has affected the demographic and socioeconomic structure of Turkey and has brought integration problems in many areas, especially in health services (1, 2).

Tuberculosis (TB) is a serious health problem in socioeconomically disadvantaged groups, especially among Syrian refugees, low-income individuals, and those living in slum areas (3). The incidence of TB is not only due to biomedical factors; it is also strongly dependent on social determinants such as poverty, poor living conditions, malnutrition, and limited access to healthcare. Effective TB control is possible not only with medical interventions, but also with multi-sectoral, holistic approaches to these social determinants (4). In regions such as southeastern Turkey, where forced migration and rapid urbanization are intense, the impact of these social inequalities on TB becomes even more pronounced.

There was a downward trend in the incidence of TB in Syria before the war (5). However, the collapse of the national health system and the interruption of vaccination and treatment services with the war brought TB control to a standstill. After the mass migration to Turkey, the share of Syrian refugees in TB cases has increased rapidly. According to the Ministry of Health TB reports, the proportion of foreign nationals among reported TB cases rose from 1.3% in 2011 to 6.8% in 2015 (6). This increase shows that the proportion of the Syrian refugee population in the TB burden has increased significantly over the years.

After the World Health Organization (WHO) declared COVID-19 a global pandemic on March 11, 2020, health systems have come under great pressure. The pandemic has led to disruptions in access to health services; it has caused delays in the diagnosis, treatment and follow-up processes of TB (7, 8). These cuts have disproportionately adversely affected vulnerable groups, especially the Syrian refugee population.

Its location close to the Turkish-Syrian border, its demographic structure directly affected by migration movements, and the increasing Syrian refugee population density over time make the assessment of the TB burden in Mardin province a critical public health priority. Although the regional burden of TB has been discussed in the existing literature, the Syrian refugee patient population has not been examined as a separate analysis. In Turkey, several studies have investigated the epidemiology of tuberculosis in the southern regions, highlighting the influence of migration and refugee populations on TB control efforts (9–11).

In this context, it is aimed to contribute to the strengthening of national and global TB control strategies by retrospectively comparing tuberculosis cases between Syrian refugees and local citizens in Mardin province between 2016 and 2023.

## 2 Materials and methods

### 2.1 Study design and ethical approval

Operational definitions and laboratory methods: In this study, “microbiological diagnosis” was defined as confirmation by smear microscopy and/or culture; “culture positivity” refers specifically to growth of *Mycobacterium tuberculosis* complex in culture. Specimens (e.g., sputum, BAL, CSF, urine, lymph node, and other tissue biopsies) were processed and analyzed in the provincial TB reference laboratory according to the National TB Program procedures, which include decontamination, concentration, AFB smear microscopy, and culture with species-level identification and first-line drug susceptibility testing as available. Referral pathways followed the routine TB dispensary registry and inter-provincial transfer system. Reporting guideline: The study was prepared in line with the STROBE statement; numbers of exclusions due to missing core variables are explicitly stated in the Results.

*Molecular testing.* Rapid molecular assays (PCR/NAAT, e.g., Xpert MTB/RIF) were not routinely or systematically available in the provincial reference laboratory; therefore, NAAT results were not captured in the registry and were not included in the primary analyses. When documented from external testing, such results were classified under microbiological confirmation.

This retrospective observational study was conducted between January 1, 2016, and December 31, 2023, using data from the Tuberculosis (TB) Dispensary Registration System in Mardin, Turkey. The study was approved by the Mardin Artuklu University Ethics Committee and the Mardin Provincial Directorate of Health (Approval No: 2024/5–10). All procedures were carried out in accordance with the principles of the Declaration of Helsinki.

### 2.2 Study groups and classification

A total of 491 patients diagnosed with TB during the study period were included in the research. Patients with missing demographic or diagnostic data were excluded from the study. (*n* = 5), as stated in accordance with the STROBE statement.

Patients were divided into two groups according to their nationality:

1. Local population: Citizens of the Republic of Turkey.
2. Syrian refugee population: Individuals of Syrian descent (regardless of whether they have official refugee status).

### 2.3 Variables and definitions

*Variables collected:*

- Demographics: age, sex, nationality, year of diagnosis, place of residence.
- BCG vaccination: presence of a BCG scar.
- Case type: new case, relapse, treatment after failure, treatment after loss to follow-up (treatment abandonment), chronic TB (disease persisting despite ≥2 completed standard regimens), and transferred-in.
- Disease localization: pulmonary, extrapulmonary, or both.
- Affected organs: lung, pleura, lymph node, bone, gastrointestinal and genitourinary systems, meninges, pericardium, skin, liver, eye, and other.
- Diagnostic methods: acid-fast bacilli (AFB) smear microscopy, histopathology, culture, and radiological findings; sample types included sputum, bronchoalveolar lavage (BAL), cerebrospinal fluid (CSF), urine, lymph-node biopsy, and abscess drainage.
- Drug resistance: susceptibility to isoniazid (INH), rifampicin (RIF), ethambutol (EMB), and streptomycin (SM); multidrug-resistant TB (MDR-TB) defined as resistance to both INH and RIF.
- Treatment outcomes: cure, treatment completion, failure, loss to follow-up (abandonment), death, or ongoing treatment.
- Directly observed treatment (DOT): DOT status as recorded in the dispensary registry.

*Operational case and outcome definitions (aligned with National TB Programme/WHO):*

New case: no prior anti-TB treatment or <1 month of treatment.

Relapse: previously declared *cured* or *treatment-completed* and diagnosed with TB again.

Treatment after failure: TB diagnosed after a prior regimen met failure criteria.

Treatment after loss to follow-up (treatment abandonment): return after ≥2 consecutive months of treatment interruption.

Transferred-in: treatment initiated/recorded in another province and continued in Mardin (administrative inter-provincial referral within the national TB system).

Transferred-out: registered in Mardin and administratively referred to another province to continue care; final outcome is recorded by the receiving unit.

Treatment success: cure + treatment completion combined (surveillance definition).

Directly observed treatment (DOT): as recorded in the dispensary registry.

*Note: “Transferred-in/out” denotes administrative inter-provincial referral and does not indicate international migration. Person-first, non-stigmatizing language was used in line with the Stop TB/WHO “Words Matter” guidance* (12).

### 2.4 TB incidence calculation

Annual TB incidence was calculated as (new TB cases ÷ mid-year population) × 100,000. Mid-year population data for local citizens were obtained from the Address-Based Population Registration System (ABPRS; NVI) (13). Refugee denominators were taken from the Presidency of Migration Management (PMM; formerly DGMM) Temporary Protection statistics and from UNHCR Operational Data Portal provincial breakdowns for Türkiye, using archived datasets covering 2016–2023 (14, 15). Refugee figures primarily reflect out-of-camp residents; temporary accommodation centres (TACs) were noted where applicable. Temporary accommodation centres (TACs) in Mardin province were largely closed by late 2016, with only a single TB case reported from a camp in early 2017; thereafter, all detected refugee TB cases represented out-of-camp residents. Year-specific mid-year snapshots were used, and minor discrepancies across sources were harmonized by prioritizing mid-year totals and applying a consistent provincial scope.

### 2.5 Statistical analysis

Descriptive statistics were summarized as frequency (%) for categorical variables and mean ± SD for age. Between-group comparisons used Pearson’s chi-square or Fisher’s exact test for categorical variables and the Mann–Whitney U test for age (two-sided *α* = 0.05). Primary analyses were descriptive. For outcomes with significant crude between-group differences (treatment success, BCG scar presence, and transferred-out during treatment), we fitted multivariable logistic-regression models with predictors group (refugee vs. local), age, and sex. Given sparse cells and the small refugee subgroup, adjusted analyses used bias-reduced (Firth) logistic regression; we report adjusted odds ratios (aORs) with 95% Wald CIs and penalized likelihood-ratio (PLR) *p*-values (all Firth models adjusted for age and sex; complete-case). Sensitivity analyses for treatment success excluded transferred-out cases and were repeated in the pulmonary-only subset. Analyses were performed in IBM SPSS Statistics 21.0 (descriptives/tests/standard logistic) and in R (logistf) for Firth regression.

## 3 Results

### 3.1 Demographic characteristics and case classification

A total of 491 tuberculosis (TB) patients, including 456 local citizens (92.8%) and 35 Syrian refugees (7.2%), were included in the study. The mean age was 39.9 years for local citizens and 37.2 years for Syrian refugees. There was no statistically significant difference in gender distribution between the two groups (*p* > 0.05), while the male–female ratio was almost equal in both.

At the time of diagnosis, 14.3% (5/35) of Syrian refugees were residing in temporary accommodation centers, while none of the local patients remained. A visible Bacillus Calmette-Guérin (BCG) scar was observed in 93.2% of the local population and 62.9% of the Syrian refugee group, a statistically significant difference (*p* < 0.05). All patients without BCG scarring were over 18 years of age. Of the local patients, 88.4% (403/456) were new TB cases, 4.6% (21/456) were recurrent cases and 6.6% (30/456) were transferred-in cases; 80.0% (28/35) of the Syrian refugees were new cases, 5.7% (2/35) were recurrences and 14.3% (5/35) were transferred-in cases. No cases of chronic or treatment failure were recorded in either group (Table 1).

**Table 1:** Demographics and case classification.

Variable	Local citizens (*n* = 456)	Syrian refugees (*n* = 35)	*p*-value
Male, *n* (%)	238 (52.2%)	18 (51.4%)	0.93 †
Female, *n* (%)	218 (47.8%)	17 (48.6%)	
Mean age (years) mean ± SD	39.9 ± 18.8	37.2 ± 19.2	0.36 ‡
Residence in temporary accommodation center, *n* (%)	0 (0.0%)	5 (14.3%)	N/A
BCG scar positive, *n* (%)*	425 (93.2%)	22 (62.9%)	<0.001 †
New case	403 (88.4%)	28 (80.0%)	0.36 †
Relapse	21 (4.6%)	2 (5.7%)	0.67 †
Transferred-in case	30 (6.6%)	5 (14.3%)	0.07 †
Total number of patients, *n* (%)	456 (92.9%)	35 (7.1%)	N/A

*p*-values from Pearson’s *χ*^2^ or Fisher’s exact test for categorical variables and from the Mann–Whitney U-test for age (two-sided). Values are *n* (%); age is reported as mean ± SD.

† Pearson’s chi-square or Fisher’s exact test (as appropriate).

‡ Mann–Whitney U-test (two-sided).

### 3.2 Disease locations and diagnostic methods

Pulmonary TB was diagnosed in 52.2% of local patients and 62.9% of Syrian refugees. Extrapulmonary involvement alone was present in 42.8% of local citizens and 31.4% of Syrian refugees, while combined pulmonary and extrapulmonary TB occurred in 4.8 and 5.7%, respectively (*p* > 0.05).

Lymph node TB was the most common extrapulmonary form in both groups, occurring in 24.1% of local citizens and 25.7% of Syrian refugees. Pleural involvement was seen in 9.9% of the local population and 5.7% of the Syrian refugees. Other forms, including gastrointestinal, bone, and genitourinary TB, were rare in both groups. Meningeal and miliary TB were reported only among local populations (four patients each, 0.9%) and were not observed among Syrian refugees. Histopathological confirmation of tuberculosis was obtained in 43.0% of local patients and 34.3% of Syrian refugee patients (*p* > 0.05). Microbiological confirmation (by microscopy or culture) was noted in 50.7% of local citizens and 62.9% of Syrian refugees. Sputum is the most commonly used diagnostic material in both groups, used in 80.5% of the local population and 96.2% of the Syrian refugees. Acid-fast bacilli (AFB) were detected in 38.8% of the local population and 48.6% of the Syrian refugees (*p* > 0.05). Culture positivity was found in 41.7% of the local population and 42.9% of the Syrian refugees with a similar distribution. Histopathology-only diagnoses (histopathology positive without microbiological confirmation recorded at registration) totaled 185/491 (37.7%) overall—173/456 (37.9%) in locals and 12/35 (34.3%) in refugees (Table 2).

**Table 2:** Disease distribution and diagnostic methods.

Variable	Local citizens (*n* = 456)	Syrian refugees (*n* = 35)	*p*-value
Pulmonary TB	239 (52.4%)	22 (62.9%)	0.42
Extrapulmonary TB	195 (42.8%)	11 (31.4%)	
Combined pulmonary + extra. TB	22 (4.8%)	2 (5.7%)	—
Lymph node TB	110 (24.1%)	9 (25.7%)	—
Pleural TB	45 (9.9%)	2 (5.7%)	—
Gastrointestinal TB	15 (3.3%)	1 (2.9%)	—
Bone TB	15 (3.3%)	1 (2.9%)	—
Meningeal TB	4 (0.9%)	0 (0.0%)	—
Miliary TB	4 (0.9%)	0 (0.0%)	—
Genitourinary TB	6 (1.3%)	0 (0.0%)	—
Histopathological diagnosis	196 (43.0%)	12 (34.3%)	0.32
Microbiological diagnosis	231 (50.7%)	22 (62.9%)	0.16
AFB positivity	177 (38.8%)	17 (48.6%)	0.17
Culture positivity	190 (41.7%)	15 (42.9%)	0.74

*p-*values are reported only for (i) the main classification (pulmonary-only vs extrapulmonary-only; combined listed separately) and (ii) diagnostic tests (histopathology, microbiology, AFB, culture) using Pearson’s *χ*^2^ or Fisher’s exact test, as appropriate. Organ-specific rows reflect any extrapulmonary involvement (including combined pulmonary + extrapulmonary); therefore, their totals do not equal the extrapulmonary-only row. Values are *n* (%).

### 3.3 Drug resistance and treatment outcomes

Resistance to at least one first-line anti-TB drug was detected in 5.7% of patients in both groups. Isoniazid (INH) resistance was found in 3.9% of local citizens and 2.9% of Syrian refugees, while rifampicin (RIF) resistance was detected in 0.9 and 2.9%, respectively. Streptomycin (SM) resistance occurred in 2.4% of local citizens and 2.9% of Syrian refugees. Ethambutol (EMB) resistance was noted in 1.5% of local patients but was not detected in Syrian refugees. Multidrug-resistant TB (MDR-TB), defined as resistance to both INH and RIF, was found in three localized patients (0.66%) and was not found in any of the Syrian refugees. Treatment was completed in 51.8% of local patients and 34.3% of Syrian refugees. Cure was achieved in 38.6% of local citizens and 34.3% of Syrian refugees. Of the local citizens, 1.3% (6 patients) discontinued treatment and 5.3% (24 patients) were transferred during treatment. No treatment abandonment was observed in the Syrian refugee group, but 25.7% (nine patients) were transferred – significantly higher than in the local population (*p* < 0.05). While Directly Observed Treatment (DOT) was applied for all local patients, one Syrian refugee patient (2.9%) was not given DOT (*p* < 0.05). Mortality rates were 3.1% in local citizens and 5.7% in Syrian refugees (Table 3).

**Table 3:** Drug resistance and treatment outcomes.

Variable	Local citizens (*n* = 456)	Syrian refugees (*n* = 35)	*p*-value
Resistance to ≥1 drug	26 (5.7%)	2 (5.7%)	1.00
INH resistance	18 (3.9%)	1 (2.9%)	0.74
RIF resistance	4 (0.9%)	1 (2.9%)	0.26
SM resistance	11 (2.4%)	1 (2.9%)	0.87
EMB resistance	7 (1.5%)	0 (0.0%)	0.45
MDR-TB (INH + RIF)	3 (0.7%)	0 (0.0%)	—
Treatment success (cure + completion)	412 (90.4%)	24 (68.6%)	0.03
Cure	176 (38.6%)	12 (34.3%)	—
Treatment completion	236 (51.8%)	12 (34.3%)	—
Abandonment of treatment	6 (1.3%)	0 (0.0%)	—
Transferred out	24 (5.3%)	9 (25.7%)	<0.001
DOT coverage	456 (100.0%)	34 (97.1%)	0.15
Mortality	14 (3.1%)	2 (5.7%)	—

Treatment success was defined as the sum of “cure” and “treatment completion.” MDR-TB was defined as resistance to both isoniazid (INH) and rifampicin (RIF). Variables with low event counts or small subgroup sizes (e.g., MDR-TB, treatment abandonment, mortality) were presented descriptively (N/A) without individual statistical comparisons.

### 3.4 Adjusted analyses of key outcomes

In Firth bias-reduced logistic regression (adjusted for age and sex), refugee status was associated with lower odds of treatment success in the full dataset (aOR 0.224, 95% CI 0.103–0.488; p_PLR < 0.01). Excluding transferred-out cases attenuated the association and rendered it non-significant (aOR 0.562, 95% CI 0.121–2.605; p_PLR = 0.42). In pulmonary-only analyses, the association persisted (aOR 0.216, 95% CI 0.083–0.567; p_PLR < 0.01). Other outcomes with crude between-group differences (BCG scar, transferred-out) are summarized in (Table 4).

**Table 4:** Firth bias-reduced logistic regression for outcomes with significant crude differences between groups.

Outcome	Events/*N* (outcome = 1)	Adjusted OR (refugee vs local)	95% CI	*p* (PLR)
Treatment success (all cases)	436/491	0.224	0.103–0.488	<0.001
BCG scar present	447/491	0.095	0.041–0.220	<0.001
Transferred-out during treatment	33/491	6.169	2.576–14.774	<0.001

Firth bias-reduced logistic regression; all models adjusted for age and sex. “Adjusted OR (refugee vs local)” compares refugees with locals (reference) at the same age and sex. *p*-values from penalized likelihood-ratio (PLR) tests; 95% Wald CIs. “Events/*N*” = outcome = 1 among complete cases; “Transferred-out” denotes inter-provincial transfer during treatment. Interpretation: OR<1 indicates lower odds in refugees; OR>1 indicates higher odds. See Methods 2.5 for details.

### 3.5 Incidence trends

The annual incidence of TB among local citizens ranged from 4.87 to 8.77 per 100,000, with an average incidence of 6.79 per 100,000 over the 8-year study period. The average incidence among Syrian refugees was 4.83 per 100,000, and there were significant fluctuations during the years of the COVID-19 pandemic. In 2020 and 2021, the incidence among Syrian refugees decreased to 0.0 and 1.09 per 100,000, respectively, while the incidence in the local population remained relatively stable. A resurgence was observed in 2022 (9.79/100,000) and 2023 (5.87/100,000), suggesting potential delays in diagnosis and access during the pandemic (Table 5).

**Table 5:** Annual number and incidence of TB cases (2016–2023).

Year	Local			Syrian refugees		
	Population	TB cases	Incidence (per 100,000)	Population	TB cases	Incidence (per 100,000)
2016	796,237	63	7.91	93,504	7	7.49
2017	809,719	71	8.77	91,172	5	5.48
2018	829,195	67	8.08	90,170	5	5.55
2019	838,778	65	7.75	89,000	3	3.37
2020	854,716	45	5.26	88,507	0	0.00
2021	862,757	42	4.87	91,455	1	1.09
2022	870,374	57	6.55	91,907	9	9.79
2023	888,874	46	5.18	85,243	5	5.87
Total		456	6.79		35	4.83

Incidence per 100,000 = cases ÷ mid-year population × 100,000. Denominators: ABPRS/NVI (locals) and DGMM/PMM and UNHCR (refugees). Year-specific mid-year snapshots (2016–2023) were used. Refugee figures reflect out-of-camp residents; temporary accommodation centers were noted where applicable. “Total” is the mean of annual incidences. No statistical comparison across years.

## 4 Discussion

This study was conducted in the Turkish province of Mardin, which borders Syria and is home to a significant Syrian refugee population, and revealed five key differences between local and Syrian refugee patients with tuberculosis (TB). First, BCG scar positivity is significantly lower in Syrian refugees than in local citizens. Second, the use of microbiological diagnostic methods falls short of the World Health Organization (WHO) targets in both groups. Third, treatment success (sum of recovery and treatment completion) is significantly lower in Syrian refugees than in local citizens. Fourthly, the cases referred to another province (“transferred-out”) during treatment were significantly higher in Syrian refugees, and the rate of cases arriving in Mardin province during treatment (“transferred-in”) was noticeably higher in the Syrian refugee population. Fifth, a dramatic decrease in the incidence of TB among Syrian refugees has been observed during the COVID-19 pandemic.

In Turkey, BCG vaccination programs are carried out in line with the World Health Organization’s (WHO) End TB strategy. According to WHO reports and literature studies, BCG vaccination rates in Turkey are around 95% (16–18). On the other hand, according to Syria’s data from the pre-civil war period, BCG vaccination was reported at a rate of 100% (19). However, in our study, a significantly lower BCG scar positivity was found in Syrian adult Syrian refugees compared to the local population. As a matter of fact, even the BCG vaccination rate of 81% reported by Syria to WHO for 2018 is well above the rates found in our study (17). A study of European countries examining the vaccination records of Syrian refugees reported that vaccination rates among Syrian refugees from the Middle East and Africa were significantly lower (20). While the BCG scar positivity rates detected in the local population were consistent with the national data declared by Turkey, the fact that this rate was significantly lower in Syrian refugees indicates the inadequacy of the tuberculosis control system in Syria since before the civil war.

In our study, microbiological diagnosis rates were slightly below the 68% target recommended by the World Health Organization in both patient groups (18, 21). This situation creates significant limitations in terms of identifying drug resistance patterns and conducting effective epidemiological follow-up. BCG scar negativity was significantly more common in the Syrian refugee population, and in this context, it is understandable that microbiological tests were studied at a higher rate in sputum samples due to the predominance of pulmonary involvement, although it is generally considered as underdiagnosis. On the other hand, the high level of BCG scar positivity in the local citizens population reduced pulmonary TB rates due to the immune-mediated protection effect; instead, it may have led to a higher rate of extrapulmonary involvement. This may explain the relatively low rates of microbiological diagnosis in domestic patients.

However, it is clear that bacteriological examinations should be expanded for both groups, especially in tissue tuberculosis. When the national data are examined, it is seen that microbiological confirmation is reported at low rates in tissue-derived TB diagnoses, similar to our study. Although the Turkish National Tuberculosis Control Program emphasizes the need to increase bacteriological diagnosis, in practice the main reason for these low rates is the priority given to pathological examinations in the diagnosis of tissue samples (9, 21). International literature points to similar problems. In a large-scale study based in China, microbiological examination was reported as 49.3% in lung TB cases and only 12.8% in tissue TB cases (22). In the other study, which was conducted in India and included only extrapulmonary tuberculosis (EPTB) cases, bacteriological positivity rate of 34% was obtained by microbiological diagnostic methods in a total of 104 cases. The main reasons for the low microbiological diagnosis are the quality of biopsies or liquid samples taken with low bacillus load in the samples and the quality of the laboratory (23). In global reports, Japan and South Korea were among the countries with low TB burden that met and even exceeded the bacteriological examination target set by WHO (24, 25). For this reason, we believe that clinical awareness trainings should be disseminated to ensure that qualified samples are sent to the laboratory for microbiological examination in all cases where tissue samples are taken.

In our study, a significant difference was found between the local population and the Syrian refugee population in Mardin province in terms of tuberculosis treatment outcomes. Although Directly Observed Treatment (DOT) was carried out at similar rates in both groups and in a way that covered the entire patient population, treatment success was found to be significantly lower in Syrian refugees. In the international literature, there are several studies and WHO reports showing that TB treatment success is lower in Syrian refugees than in the local population (4, 18, 26). It is suggested that this difference cannot be explained only by the application of DOT, but may be related to socioeconomic and structural factors such as the lack of a settled residence of Syrian refugee individuals and their frequent relocation (5, 26–29). The review report published by Pareek et al., which identified the lack of a settled life of Syrian refugees in high-income European countries and reinfection with TB during travels to their homeland as a risk factor, was found to be related to the temporary return of the Syrian refugee population in our study to Syria, especially during religious holidays (27). In addition to the risk factor covering border crossing, in our study, the rate of “transferred-in” and “transferred-out” of Syrian refugees within the country was found to be noticeably higher than the local citizens. In the literature and reports, it has been stated that this relocation may lead to interruption or interruption of the treatment process, and at the same time, it negatively affects contact control, which has a very important place in the control of latent TB infection (20, 27–29). Similarly, in our study, low treatment success in Syrian refugees may be related to social determinants such as relocation and non-sedentary lifestyle, despite the adequacy of DOT administration.

In recent years, as in many countries, decreases in the incidence of TB have been reported in Turkey as well as in the fight against TB. However, there is no reported decrease in the incidence of TB in the Syrian refugee population in the world, and an increase in the incidence of TB in Syrian refugees is reported in the data of Turkey. This indicates that TB has increasingly affected Syrian refugees as a consequence of migration-related vulnerabilities (20, 27–29). In our results, although the TB incidence data were examined on the basis of local and limited number of patients, it remained at lower rates in the Syrian refugee population than in the domestic population in order to give an idea. This situation suggests the possibility of Syrian refugees encountering under-diagnosis in Mardin. The socio-cultural and demographic structure of Mardin province is suitable for the accommodation of Syrian refugees in the local population and even for their cohabitation due to kinship relations (30, 31). It is a well-known fact that TB cases may hesitate to resort to diagnosis and treatment in order not to be exposed to the stigma they may encounter in the social and even health environment (32, 33). The relatively low incidence rates of Syrian refugees observed in Mardin province remained low according to both Turkey data and the incidence data of Syrian refugee TB cases in the world (6, 25, 28, 29). This can be explained by the behavior of not seeking medical help in tuberculosis to avoid social stigma and even for fear of deportation. In the literature, it has been stated that Syrian refugees may exhibit similar behaviors due to fear of social stigma and deportation in the countries they live in (20, 34).

The most prominent data of our study in incidence rates was the abnormal decrease in the incidence of TB in the Syrian refugee population in 2020 and 2021, the years of the COVID-19 pandemic. In the subsequent TB incidences in 2022, an increase was observed in the Syrian refugee population compared to the local population. These findings show that the Syrian refugee population was deprived of health services during the pandemic years, especially in terms of TB diagnosis. Studies have shown that pandemic-specific changes in health systems around the world during the COVID-19 pandemic have resulted in a weakening of TB diagnosis and follow-up chains in Syrian refugee populations, resulting in fewer diagnoses of Syrian refugees and an increased TB burden in the Syrian refugee population (7, 8, 35). As a matter of fact, when the post-pandemic data were examined in our study, the increase in the incidence of TB in Syrian refugees was remarkable. However, despite this, due to the reasons discussed above; societal stigma and fear, and even deprivation of immigration rights, etc.; in the province of Mardin, we think that the Syrian refugee population is still experiencing under-diagnosis. For this reason, TB control programs need to be handled in a confidentiality-assured, anti-stigma, culturally sensitive and accessible structure for Syrian refugees. Studies carried out on a national basis will contribute to revealing this problem more clearly in the future.

## 5 Conclusion

This study demonstrates significant differences between local citizens and Syrian refugees with TB in Mardin – a border province with substantial cross-border mobility. Lower BCG scar rates, reduced treatment success, and higher transfer-out rates among refugees highlight challenges in treatment continuity and follow-up. Both groups showed microbiological diagnosis rates below WHO targets, emphasizing the need to expand bacteriological confirmation, particularly for extrapulmonary TB.

Although social cohesion between refugees and locals may appear advantageous, fear of stigmatization may discourage refugees from seeking diagnosis and treatment.

Therefore, TB control in border regions should incorporate refugee-sensitive strategies, including:

- confidential and stigma-free access to healthcare,
- strong inter-provincial follow-up systems to prevent treatment interruption due to relocation, and
- improved laboratory capacity to ensure timely bacteriological confirmation.

Such approaches may strengthen treatment adherence and prevent transmission in mobile and vulnerable populations.

## Data Availability

The raw data supporting the conclusions of this article will be made available by the authors, without undue reservation.

## References

[1] Mülteciler ve Sığınmacılar Yardımlaşma ve Dayanışma Derneği (2023). Türkiye’deki Suriyeli Sayısı Haziran 2023. Available online at: https://multeciler.org.tr/turkiyedeki-suriyeli-sayisi-haziran-2023/ (Accessed June 9, 2025).

[2] McAuliffe M Ruhs M. World migration report 2018. Geneva: International Organization for Migration (IOM) (2017).

[3] Litvinjenko S Magwood O Wu S Wei X. Burden of tuberculosis among vulnerable populations worldwide: an overview of systematic reviews. Lancet Infect Dis. (2023) 23:1395–407. doi: 10.1016/S1473-3099(23)00372-9, PMID: 37696278 PMC10665202

[4] Kasaeva T Mavhunga F Viney K Dias HM van den Boom M Severoni S . Innovative solutions for the elimination of tuberculosis among refugees and migrants. Geneva: World Health Organization (2024).

[5] Ismail MB Rafei R Dabboussi F Hamze M. Tuberculosis, war, and refugees: spotlight on the Syrian humanitarian crisis. PLoS Pathog. (2018) 14:e1007014. doi: 10.1371/journal.ppat.1007014, PMID: 29879218 PMC5991642

[6] T.C. Sağlık Bakanlığı Halk Sağlığı Genel Müdürlüğü (2017). Türkiye’de Verem Savaşı 2017 Raporu. Ankara: T.C. Sağlık Bakanlığı.

[7] Turan H Gunak F Yasaci Z Ethemoglu G Aygun S. Impact of the COVID-19 pandemic and migration on tuberculosis notifications: a retrospective analysis with 5-year data from three centers. Eur J Clin Microbiol Infect Dis. (2024) 43:2001–9. doi: 10.1007/s10096-024-04918-4, PMID: 39110338

[8] Migliori GB Thong PM Alffenaar J-W Denholm J Tadolini M Alyaquobi F . Gauging the impact of the COVID-19 pandemic on tuberculosis services: a global study. Eur Respir J. (2021) 58:2101786. doi: 10.1183/13993003.01786-2021, PMID: 34446465 PMC8581650

[9] Kabak M Çil B Hocanlı I Sezgi C Taylan M Düzenli U. The retrospective evaluation of tuberculosis cases in Mardin between 2012 and 2018. Eastern J Med. (2019) 24:330–4. doi: 10.5505/ejm.2019.49368

[10] Department of Chest Diseases, Harran University School of Medicine, Şanlıurfa, Turkey, Kurtuluş Ş Can R, Nursing Public Health, Mustafa Kemal Atatürk Vocational and Technical Anatolian High School, Eskişehir, Turkey. The effect of environmental exposures on the diagnosis of tuberculosis in Syrian refugees. Turk Thorac J (2021) 22:489–493. doi: 10.5152/TurkThoracJ.2021.21158 35110266 PMC8975346

[11] Ergönül Ö Tülek N Kayı I Irmak H Erdem O Dara M. Profiling infectious diseases in Turkey after the influx of 3.5 million Syrian refugees. Clin Microbiol Infect. (2020) 26:307–12. doi: 10.1016/j.cmi.2019.06.022, PMID: 31284037 PMC7129060

[12] Stop TB Partnership. Words matter language guide. Available online at: https://www.stoptb.org/words-matter-language-guide (Accessed June 9, 2025).

[13] T.C. İçişleri Bakanlığı, Nüfus ve Vatandaşlık İşleri Genel Müdürlüğü. Adres Kayıt Sistemi (AKS). Available online at: https://www.nvi.gov.tr/adres-kayit-sistemi (Accessed June 9, 2025).

[14] Republic of Türkiye, Ministry of Interior – Presidency of Migration Management (PMM). Temporary protection – statistics. Available online at: https://en.goc.gov.tr/temporary-protection27 (Accessed June 9, 2025).

[15] UNHCR Turkey. Turkey: Syrian Refugee Camps Provincial Breakdown (2016). Available online at: https://data.unhcr.org/en/documents/details/51789 (Accessed June 9, 2025)

[16] Yavuz T Arbak P Öztürk CE Kocabay K. BCG vaccination: where are we now? Tuberk Toraks. (2004) 52:47–51. PMID: 15143372

[17] World Health Organization. Immunization and vaccine-preventable communicable diseases. Available online at: https://www.who.int/data/gho/data/themes/immunization ([Accessed June 9, 2025)

[18] World Health Organization. Global Tuberculosis Report 2022. Geneva: World Health Organization (2022).

[19] World Health Organization. Bacillus Calmette–Guérin (BCG) vaccination coverage. Available online at: https://immunizationdata.who.int/global/wiise-detail-page/bacillus-calmette-gu%C3%A9rin-%28bcg%29-vaccination-coverage (Accessed June 5, 2025)

[20] Boudville DA Joshi R Rijkers GT. Migration and tuberculosis in Europe. J Clin Tuberc Other Mycobact Dis. (2020) 18:100143. doi: 10.1016/j.jctube.2020.100143, PMID: 31956700 PMC6957817

[21] T.C. Sağlık Bakanlığı – HSGM. Tüberküloz Rehberleri. Available online at: https://hsgm.saglik.gov.tr/tr/dokumanlar-tuberkuloz/rehberler.html (Accessed May 26, 2025)

[22] Pang Y An J Shu W Huo F Chu N Gao M . Epidemiology of Extrapulmonary tuberculosis among inpatients, China, 2008–2017. Emerg Infect Dis. (2019) 25:457–64. doi: 10.3201/eid2503.180572, PMID: 30789144 PMC6390737

[23] Nishal N Arjun P Arjun R Ameer KA Nair S Mohan A. Diagnostic yield of CBNAAT in the diagnosis of extrapulmonary tuberculosis: a prospective observational study. Lung India. (2022) 39:443–8. doi: 10.4103/lungindia.lungindia_165_22, PMID: 36629205 PMC9623858

[24] World Health Organization. Global tuberculosis report 2019. Geneva: World Health Organization (WHO) (2019). Available online at: https://www.who.int/publications/i/item/global-tuberculosis-report-2019 (Accessed May 28, 2025).

[25] World Health Organization. Global tuberculosis report 2020. Geneva: World Health Organization (2020).

[26] Legesse T Admenur G Gebregzabher S Woldegebriel E Fantahun B Tsegay Y . Tuberculosis (TB) in the refugee camps in Ethiopia: trends of case notification, profile, and treatment outcomes, 2014 to 2017. BMC Infect Dis. (2021) 21:139. doi: 10.1186/s12879-021-05828-y, PMID: 33535974 PMC7856765

[27] Pareek M Greenaway C Noori T Munoz J Zenner D. The impact of migration on tuberculosis epidemiology and control in high-income countries: a review. BMC Med. (2016) 14:48. doi: 10.1186/s12916-016-0595-5, PMID: 27004556 PMC4804514

[28] Meaza A Tola HH Eshetu K Mindaye T Medhin G Gumi B. Tuberculosis among refugees and migrant populations: systematic review. PLoS One. (2022) 17:e0268696. doi: 10.1371/journal.pone.0268696, PMID: 35679258 PMC9182295

[29] World Health Organization. Title: WHO releases new report addressing TB among refugees and migrants (2024). Available online at: https://www.who.int/news/item/19-11-2024-who-releases-new-report-addressing-tb-among-refugees-and-migrants (Accessed May 31, 2025).

[30] Apak H. Adaptation and future expectations of Syrian refugees living in Mardin: comparison between 2014 and 2021. İçtimaiyat. (2022) 6:252–68. doi: 10.33709/ictimaiyat.1055490

[31] The Guardian. ‘We feel safe here’: Historic Turkish tourist city opens its doors to Syrian quake survivors. (2023) Available online at: https://www.theguardian.com/global-development/2023/mar/09/mardin-historic-turkish-tourist-city-syrian-quake-survivors (Accessed May 31, 2025)

[32] Smith A Herington E Loshak H. Tuberculosis stigma and racism, colonialism, and migration: A rapid qualitative review. Ottawa, ON: Canadian Agency for Drugs and Technologies in Health (CADTH) (2021). 34260166

[33] Bodur MS Çil B. A research on healthcare professionals’ stigma towards tuberculosis patients. Thorac Res Pract. (2025) 26:88–96. doi: 10.4274/ThoracResPract.2024.24079, PMID: 39930730 PMC12047200

[34] Woldesemayat EM. Tuberculosis in migrants is among the challenges of tuberculosis control in high-income countries. Risk Manag Healthc Policy. (2021) 14:2965–70. doi: 10.2147/RMHP.S314777, PMID: 34285610 PMC8285291

[35] Palanca PA Rodriguez-Morales AJ Franco OH. The impact of the COVID-19 pandemic on tuberculosis services. Int J Mycobacteriol. (2021) 10:478–9. doi: 10.4103/ijmy.ijmy_223_21, PMID: 34916472

